# Intermittent theta burst stimulation modulates biceps brachii corticomotor excitability in individuals with tetraplegia

**DOI:** 10.1186/s12984-022-01049-9

**Published:** 2022-07-17

**Authors:** Neil Mittal, Blaize C. Majdic, Carrie L. Peterson

**Affiliations:** Biomedical Engineering, College of Engineering, Rehabilitation Engineering to Advance Ability Lab, Virginia Commonwealth University, Biotech Eight, 737 N 5th Street, Richmond, VA 23219 USA

**Keywords:** Transcranial magnetic stimulation, Motor evoked potentials, Spinal cord injury, Neuromodulation, Rehabilitation

## Abstract

**Background:**

Intermittent theta burst stimulation (iTBS) is a form of repetitive transcranial magnetic stimulation (TMS) that can increase corticomotor excitability of hand muscles in individuals with spinal cord injury (SCI). The objective of this study was to determine the effect of iTBS on the corticomotor excitability of the biceps brachii in individuals with tetraplegia.

**Methods:**

Ten individuals with low cervical SCI (C5-C8) and ten nonimpaired individuals completed three independent sessions. Motor evoked potentials (MEPs) served as our measure of corticomotor excitability and were collected before and after iTBS. MEPs were normalized by the electromyography corresponding to maximum voluntary contraction and analyzed using linear mixed effects models to determine the effect of iTBS (active or sham) on normalized MEPs (nMEPs). iTBS effects were compared to a ratio of active and resting motor thresholds as a measurement of corticomotor conductance potential.

**Results:**

Relative to sham, active iTBS increased nMEPs over time (*p* < 0.001) in individuals with SCI, but not nonimpaired individuals (p = 0.915). The amplitude of nMEPs were correlated with the biceps corticomotor conductance potential (p < 0.001), with nMEPs decreasing as the ratio increased at different rates after sham or active iTBS.

**Conclusions:**

Preliminary results suggest that iTBS increases biceps corticomotor excitability in individuals with tetraplegia with effects that may be predicted by corticomotor conductance potential.

*Clinical trial registration* NCT03277521 Registered on clinicaltrials.gov on August 24, 2017

**Supplementary Information:**

The online version contains supplementary material available at 10.1186/s12984-022-01049-9.

## Introduction

Spinal cord injury (SCI) often results in deficits in voluntary control of muscles due to injury induced necrosis and partial or complete loss of conduction in neural pathways. The most common neurological classification of SCI is tetraplegia, which results from injury to the cervical spinal cord and is characterized by deficits in upper and lower limb function [1, 2]. Upper limb function is the most important resource for individuals with tetraplegia [3]. Thus, improving upper limb function is a crucial part of rehabilitation to enhance an individual’s independence and quality of life. One approach to improve voluntary control of upper limb muscles is to strengthen the connection of spared corticospinal tracts through repetitive transcranial magnetic stimulation (rTMS) [4–6]. High frequency (i.e., > 5 Hz) rTMS can increase corticospinal and primary motor cortex (M1) excitability [7]. Several studies have applied rTMS over the arm and leg motor representations in the M1 in nonimpaired individuals and in patients with motor impairments to increase corticospinal and M1 excitability, voluntary motor control, and motor learning processes [8–11]. Although the effectiveness using different forms of rTMS in nonimpaired individuals and patients with motor impairments are variable [5, 8, 12, 13], rTMS may represent a useful technique to improve upper limb function after SCI, particularly when paired with other therapies.

A greater understanding of the utility of rTMS to improve upper limb function after SCI is needed. High-frequency rTMS protocols have been tested in individuals with tetraplegia to improve upper limb motor and sensory function in five studies to date, all of which targeted stimulation to hand representations in the M1 [4, 9, 14–16]. Five sessions of rTMS alone (i.e., without adjunct therapy) improved hand motor and sensory function in one study [14]. However, in a larger study involving five sessions of rTMS, results showed only modest improvement in hand motor and sensory function, which was not statistically different from sham effects, and there was no change in clinical neurological assessment [4]. Only two studies have evaluated a more specific pattern of rTMS known as intermittent theta-burst stimulation (iTBS) targeting the upper limb in individuals with tetraplegia [9]; these studies demonstrated safety and feasibility [16], and modifiability of corticomotor excitability [9]. iTBS has gained much interest, arguably due to its efficacy, short stimulation period, and effects lasting up to 60 min post-stimulation [17], making iTBS well suited as a neural priming adjunct to motor training exercises.

Further research is needed to investigate the potential for iTBS to increase the excitability of the corticospinal motor system (hereafter referred to as corticomotor excitability) in individuals with tetraplegia. Effects of iTBS have been demonstrated primarily in nonimpaired humans with stimulation applied to hand representations in the M1 and motor-evoked potentials (MEPs) recorded from the first dorsal interosseous [17–20]. A meta-analysis of studies in nonimpaired participants found that iTBS applied for 190 s significantly increases corticomotor excitability, as measured by MEPs, lasting up to 60 min with a mean maximum potentiation of 35.54 ± 3.32% [17]. The mechanisms of these effects are believed to be due to changes in neural circuits in the cortex, perhaps involving long-term potentiation of cortical synapses [21, 22]. Evidence from SCI studies in rats suggests that iTBS is able to facilitate MEPs and improve forelimb motor function after injury [23, 24], consistent with the mechanistic understanding of iTBS [21, 22]. However, Fassett et al. [25] investigated the effects of iTBS on corticomotor excitability of the flexor carpi radialis in humans with cervical SCI and found corticomotor excitability (i.e., MEPs) to be reduced in the majority of instances after a single session of active M1 stimulation. While the results of Fassett et al. contradict previous findings in nonimpaired subjects and animal models of SCI, the results indicate that iTBS is able to modify corticomotor excitability in humans with tetraplegia, which warrants further investigation.

Depending on the specific injury and needs of an individual with tetraplegia, the biceps brachii may be responsive to iTBS and a functionally relevant target for rehabilitation. The biceps may be particularly responsive to iTBS in individuals with tetraplegia because: the biceps typically remains with some spared motor pathways and function after injury at or below C6 as the biceps is primarily innervated at the C5 and C6 levels [26], and biceps motoneurons receive more corticospinal monosynaptic facilitation relative to its antagonist [27, 28]. Additionally, the biceps is relevant for upper limb rehabilitation in tetraplegia as the biceps can be transferred to restore elbow extension for some individuals with tetraplegia [29, 30]. In our previous work, we found a positive relationship between the corticomotor excitability of the transferred biceps and elbow extension strength, suggesting that increased biceps corticomotor excitability may improve the outcomes of tendon transfer surgery [31].

We present a sham-controlled pilot study to provide the first characterization of iTBS-induced effects targeting the biceps brachii in individuals with tetraplegia. The purpose of this study was to determine the effect of iTBS on corticomotor excitability of the biceps in individuals with tetraplegia and nonimpaired subjects. The nonimpaired control group is included to provide a context for the potential effects of iTBS in individuals with SCI. We hypothesized that biceps corticomotor excitability, as measured by MEPs, would be increased relative to baseline following active iTBS relative to sham iTBS in both subject groups. This hypothesis was based on the expectation that iTBS promotes long-term potentiation within cortical neurons. Since the effects of iTBS can be variable across sessions [8, 13, 32], we tested participants across three sessions to evaluate the reproducibility of iTBS aftereffects.

## Methods

### Participants

Ten individuals (8 men, 2 women) with cervical SCI aged between 23 and 53 years (mean age = 35.7 years, standard deviation = 13 years) completed this pilot study. SCI participant characteristics are provided in Table 1. Inclusion criteria required SCI participants to be between the ages of 18 and 65 years old and have an injury to the lower cervical spinal cord (C5-C8) at least 1 year prior to the date of participation. Exclusion criteria included presence of concurrent severe medical illness, including unhealed decubiti, use of baclofen pumps, existing infection, cardiovascular disease, significant osteoporosis, history of pulmonary complications, or any contraindication to TMS. Ten nonimpaired individuals (5 men, 5 women), aged between 18 and 38 years (mean age = 25.3 years, standard deviation = 5.6 years) also participated. Nonimpaired individuals with active motor thresholds (AMT) greater than 71% of maximum stimulator output (MSO) during the first assessment were excluded. This criterion was needed to ensure iTBS could be delivered at 80% of AMT by the stimulator, as the iTBS stimulation intensity was limited to a maximum of 57% MSO as a manufacturer safety feature. All participants were screened to ensure safety of the TMS protocols and provided informed consent. The protocol was approved by the Institutional Review Board of Virginia Commonwealth University.

**Table 1:** Demographic and injury information from all participants with spinal cord injury are shown

ID	Age	Gender	Injury level	Years post-injury	ISNCSCI score
1	23	M	C5-C6	7	C
2	26	M	C7	2	B
3	25	M	C5	2	A
4	42	M	C6	5	D
5	29	M	C5	4	A
6	52	F	C6	15	A
7	53	M	C6	17	A
8	32	M	C5-C6	9	B
9	30	M	C5-C6	4	A
10	52	F	C5-C8	10	D

M: male; F: female; C: cervical level injury (i.e. C5); ISNCSCI, International Standards for Neurological Classification of Spinal Cord Injury (A = no motor or sensory function is preserved in the sacral segments; B = sensory function is preserved below the level of injury, but no motor function; C = motor function is preserved below the level of injury, more than half the muscles have a grade < 3; D = motor function is preserved below the level of injury, at least half the muscles have a grade ≥ 3; E = motor and sensory function are normal)

**Fig. 1:**
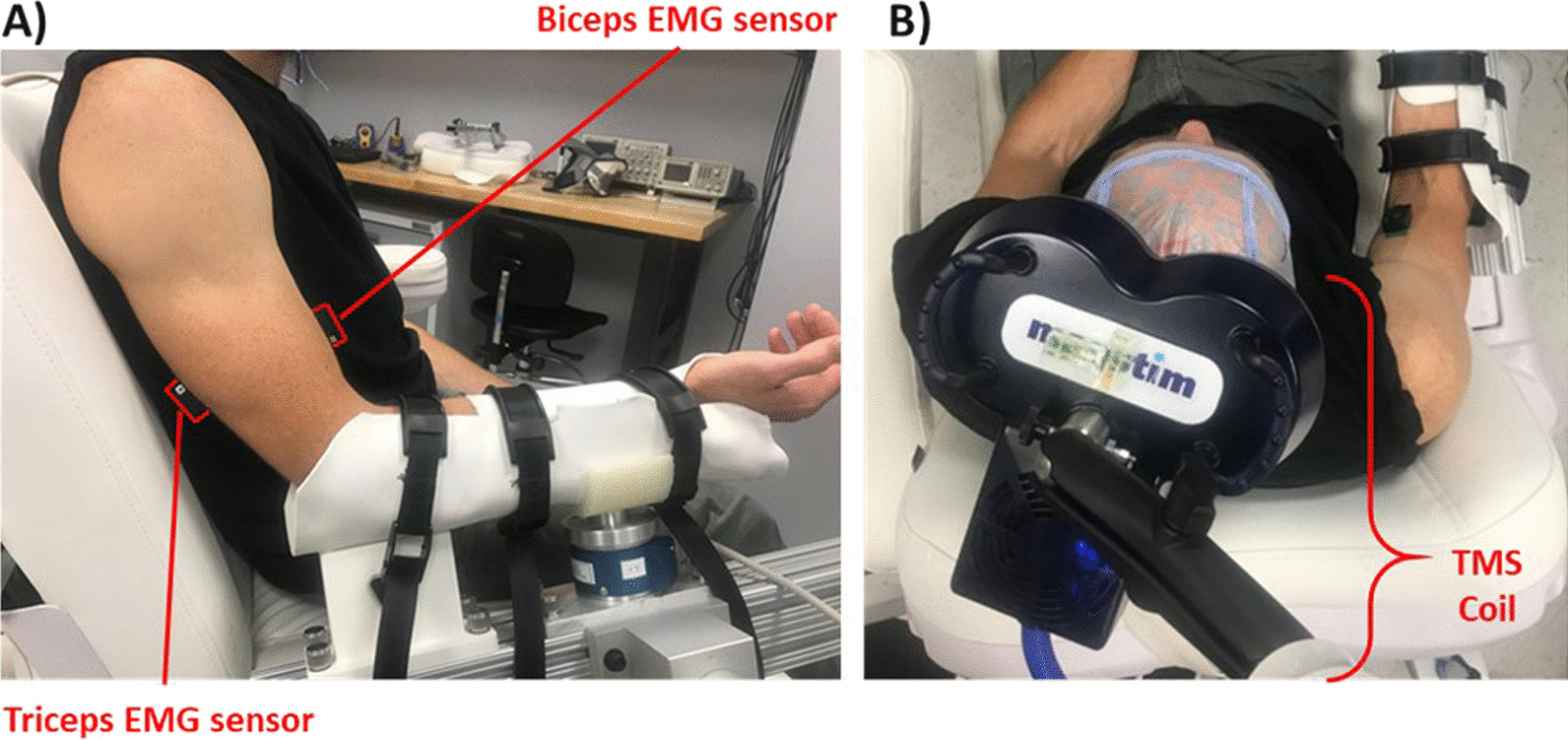
Setup for iTBS sessions. **A)** Participants’ forearms were supported in the horizontal plane with EMG sensors on the biceps and triceps; **B)** The TMS coil was placed tangentially over the scalp above the biceps representation of the motor cortex, oriented to induce a biphasic posterior-anterior then anterior–posterior current within in the motor cortex

Each participant completed three independent sessions of the iTBS protocol, yielding 30 independent sessions in the nonimpaired group, and 30 independent sessions in the SCI group. This number of sessions was established through statistical consultation and was similar to a previous study that investigated continuous TBS [32]. Repeated sessions were conducted to investigate independence of sessions and intrasubject variability, similar to previous work [8, 13]. Each session was separated by a minimum of 3 days to prevent the potential for carry over effects from one session to another [12]. To control for variability that may result from diurnal effects, sessions were scheduled for early afternoons. In each session, participants were seated in a chair with their dominant arm at rest, the elbow in 90° flexion, and the forearm supinated (Fig. 1). During portions of the protocol involving TMS, participants wore a neck brace to minimize head movements.

### Experimental protocol

**Fig. 2:**
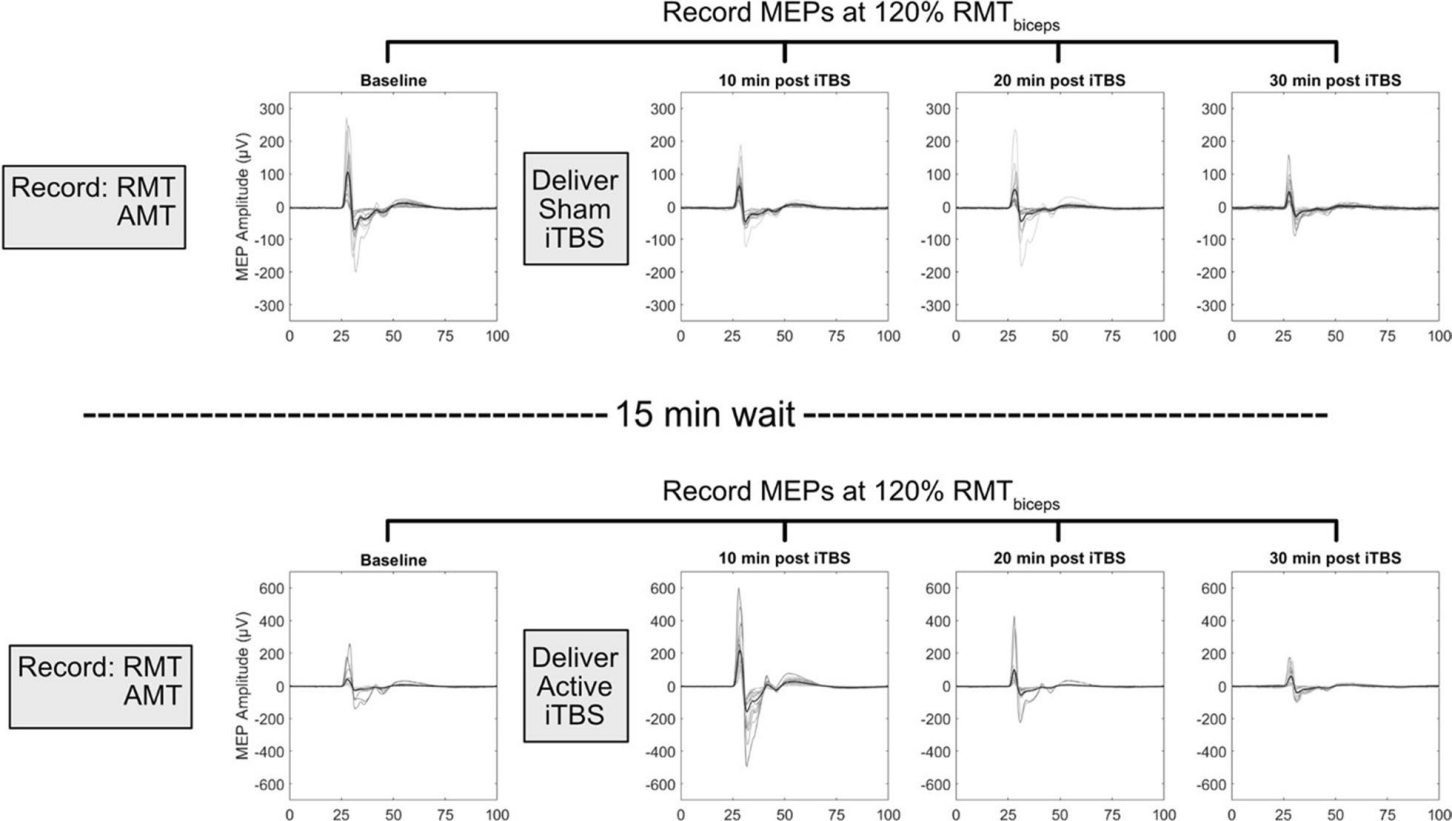
Experimental design of each session. Before each application of iTBS, single pulse TMS was used to determine RMT, AMT, and collect baseline MEPs for the biceps. The intensity of iTBS was set to 80% of AMT. Single pulse TMS was used to record MEPs at 10-min intervals following iTBS, at an intensity of 120% RMT. Data shown represent the processed and collected raw MEPs of a single session from a representative participant. Grey lines represent individuals MEPs and the black line represents the average MEP. Horizontal axis depicts time post single pulse TMS (ms)

At the beginning of each session, the biceps resting motor threshold (RMT), active motor threshold (AMT), and baseline corticomotor excitability (MEPs prior to iTBS) were recorded (Fig. 2). iTBS was then delivered, after which MEPs were recorded at intervals 10, 20, and 30 min post-iTBS (Fig. 2). This process was performed for both sham and active iTBS with participants receiving a 15-min break in between. Sham iTBS was always performed prior to active iTBS to prevent the possibility of effects from active iTBS lingering throughout the sham portion of the study. Participants were blinded to the stimulation type.

### Electromyography

Electromyography (EMG) data were recorded from the long head of the biceps and lateral head of the triceps (for monitoring) of the dominant arm of each participant using a Trigno™ Wireless System (Delsys, Natick, MA). Surface EMG electrode placement was verified by functional muscle testing. EMG signals were amplified (× 1000), bandpass-filtered (20–450 Hz) prior to A/D conversion (Micro 1401 MkII, Cambridge Electron Design, Cambridge, UK), and sampled at 2000 Hz with Spike 2 software (Cambridge Electron Design, Cambridge, UK).

### Single pulse transcranial magnetic stimulation

Single pulse TMS of the motor cortex was applied opposite to the resting arm using a Super Rapid^2^ Plus^1^ stimulator (Magstim, Whitland, UK) via a 70 mm figure-of-eight coil (P/N 3910-00). To better simulate a clinical environment where likely only one stimulation device would be available, this stimulator was used to deliver single pulse TMS and repetitive iTBS. The vertex at the intersection of the inion-nasion and inter-aural lines were marked on a fitted cap and used to identify the starting point for the coil center, 5 cm from the vertex and rotated 45 degrees from the midline. The coil was held tangentially on the scalp via a support stand. The exact hotspot for the biceps was identified (and marked on the participants cap) as the coil location and orientation evoking the largest peak-to-peak amplitude MEP using the lowest stimulation intensity from a biphasic current oriented posterior to anterior then anterior to posterior across the central sulcus.

### Motor thresholds and corticomotor excitability

Resting and active motor thresholds were determined as the lowest stimulus intensity that induced MEPs in at least 5 of 10 consecutive stimuli, either at rest (RMT) and of ≥ 50 µV with the biceps fully relaxed, or with muscle activity (AMT) and of ≥ 200 µV [33]. Muscle activity was generated by sustained isometric contraction of 10 ± 5% of the participant’s maximum effort [34]. Maximum effort was measured by the average EMG in the highest 0.5 s period of a 5 s isometric maximum voluntary contraction (MVC), averaged across 3 trials. Thresholds were found via validated adaptive parameter estimation by sequential testing software [35]. Evoked Potential Operant Conditioning Software developed and shared by the National Center of Neuromodulation for Rehabilitation was used to record motor thresholds and display effort levels for participants.

### Intermittent theta burst stimulation protocol

iTBS was applied using a Magstim Super Rapid^2^ Plus^1^ stimulator and Magstim 70 mm figure-of-eight double air film coil (3910–00) following a protocol [18] commonly applied to motor areas [8, 13, 32, 36]. iTBS comprised three pulses at 50 Hz, repeated every 200 ms for 2 s at an intensity of 80% of the participant’s AMT [17, 18]. Two second bursts were repeated every 8 s for a total of 600 pulses [18]. During sham iTBS, a Magstim 70 mm figure-of-eight double air film sham coil (3950–00) was used which looked and sounded identical to the active coil without delivering stimulation. Participants were blinded to the type of stimulation they were receiving.

### Data processing

For each session, peak-to-peak MEP amplitudes in response to single pulse TMS were extracted from the biceps EMG data using purpose-written code (MATLAB v 9.7.0.1190202). The root mean square (RMS) amplitude was calculated over a 50 ms window for the evoked response (starting 12–62 ms after the TMS pulse), and a 50 ms window prior to the TMS pulse (pre-stimulus). Instances where the pre-stimulus RMS amplitude was greater than the evoked response RMS amplitude, or where voluntary activation was detected, were discarded [37]. MEP amplitudes were then normalized by, and are presented as a percentage of, the recorded EMG MVC. Normalized MEPs (nMEPs) served as our measure of corticomotor excitability, with the average of nMEPs collected prior to iTBS serving as the baseline.

### Statistical analyses

The effects of iTBS on nMEPs were analyzed with linear mixed effects models (LMEM) using purpose-written R code based on the LME4 package [38, 39]. The model had a nested random effect of session within participant to account for potential relationships between nMEPs of the same session or participant, and within each time period post-iTBS. Coil (i.e., active or sham), time (i.e., 10, 20 or 30 min post-iTBS), and their interaction were included as fixed effects to investigate the difference in nMEPs between baseline and post-iTBS, and the differences in post-iTBS nMEPs after active or sham stimulation. A Kenward-Rogers adjustment was used to adjust for estimated random effect parameters [40]. To investigate the effect of repeated sessions and confirm the independence of sessions of the same participant, we repeated our LMEM with sessions as a fixed effect. To establish any differences between the populations’ baseline excitability, baseline metrics (RMT, AMT, and baseline nMEPs) were also compared between the nonimpaired and SCI groups using a two-tailed Mann–Whitney test.

### Corticomotor conductance potential

The biceps AMT/RMT ratio (i.e., AMT of the biceps divided by RMT of the biceps) was evaluated within a linear mixed effects model to assess a main effect, and interactions with time and type of stimulation, to account for the effects of corticomotor conductance potential on nMEPs. By corticomotor conductance potential, we refer to the synaptic conductance gradient between different states of activation along the corticospinal pathway being stimulated during a given session [41, 42]. Motor thresholds reflect this conductance as they are determined by the synaptic permeability between neurons along the corticomotor tract at rest (RMT) and during activation (AMT) [34, 43]. Therefore, we defined the biceps AMT/RMT ratio as a representation of the corticomotor conductance potential across states of activation. We evaluated the effect of corticomotor conductance on nMEPs because nMEPs represent instantaneous corticomotor excitability driven by shifts in sodium channel currents and are affected by gamma aminobutyric acid receptor modulation [41, 42].

### Post hoc analysis

In most sessions (25 out of 30), due to RMT values being greater than 84% MSO in at least one RMT measurement, we were unable to record MEPs at stimulus intensities of 120% of RMT, introducing possible under-stimulation increased MEP variability [36]. Thus, we evaluated if the nMEP amplitudes were dependent on RMT using the aforementioned LMEM with RMT as a fixed effect.

## Results

### Availability of data and materials

The dataset supporting the conclusions of this article is available in the Open Science Framework (https://osf.io/za78p/?view_only=1f23b70066d64faba087b2b4c0784baa).

### Change in normalized MEPs post-iTBS

**Fig. 3:**
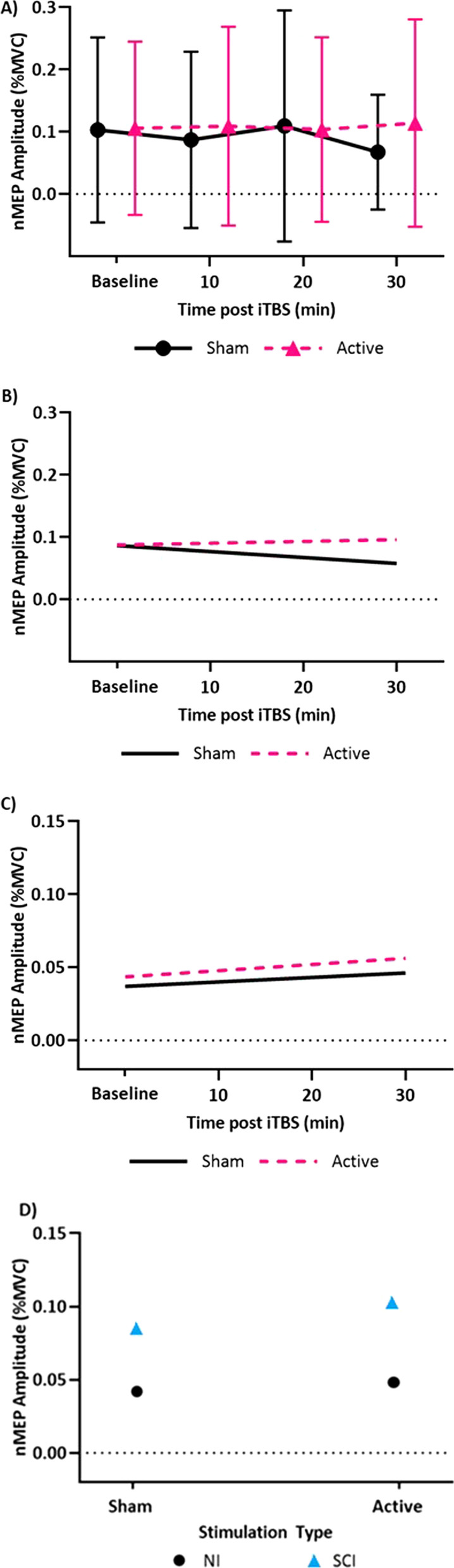
Time differentiated normalized motor evoked potential amplitudes (nMEP). **A)** Mean of recorded nMEP amplitudes for each time point across all 30 sessions for active and sham iTBS are presented for participants with SCI. Error bars represent one standard deviation from the mean. **B)** In the SCI group, the linear mixed effects model (LMEM) shows a significant difference over time in nMEP amplitudes depending on the type of iTBS, active or sham. **C)** In the nonimpaired group, the LMEM does not show an effect of stimulation type on nMEP amplitude. **D)** There was a difference in the effect of iTBS between groups, based on the LMEM, consistent with the excitation seen in the SCI group and not seen in the nonimpaired group. Each point represents all nMEPs across all sessions, for the given group and stimulation type

In individuals with SCI, there was an effect of active iTBS relative to sham stimulation over time (p < 0.001, χ^2^ = 18.6) with active iTBS causing an increase in nMEPs from baseline. For each time point, the average nMEP amplitude is presented in Fig. 3A. Modeled nMEPs resulting from the LMEM are presented in Fig. 3B for both the active and sham conditions. In nonimpaired individuals, change in nMEPs from baseline did not differ for the active and sham conditions as indicated by no interaction between the type of stimulation and time post-iTBS (p = 0.915) in the analysis of the LMEM (Fig. 3C). When comparing the SCI group to the nonimpaired group, there was an interaction between group and stimulation type within the LMEM (p = 0.012, χ^2^ = 6.4) (Fig. 3D).

nMEPs were independent of session, suggesting no carryover effects and no relationship between sessions within a participant (p = 0.074, χ^2^ = 3.2).

With regards to group baseline metrics, there was a difference in baseline nMEPs (p < 0.001) between the nonimpaired and SCI groups. There was no difference between the two groups with respect to MVC EMG (p = 0.90), RMT (p = 0.081), AMT (p = 0.50), or motor threshold ratio (p = 0.89). Group average baseline metrics are provided in Table 2. Individual participant motor thresholds and MVC values can be found in the Additional file 1: Tables S1, S2, and S3.

**Table 2:** Baseline biceps metrics for the nonimpaired and SCI groups

	Nonimpaired	Spinal cord injury
MVC EMG (mV)	274 ± 12	250 ± 18
RMT (%MSO)	88.5 ± 11	92.1 ± 11
AMT (%MSO)	57.3 ± 8	66.4 ± 21
AMT/RMT Ratio	0.67 ± 0.1	0.69 ± 0.2
Baseline nMEP *	0.0403 ± 0.041	0.1031 ± 0.148

Data presented by means within the group across all sessions and the standard deviation (mean ± std). (*) Represents significant difference (*p* < 0.05) between groups. The AMT/RMT ratio represents the corticomotor conductance potential

### Corticomotor conductance potential

In the SCI group, there was a significant interaction between the biceps AMT/RMT ratio (i.e., corticomotor conductance potential) and stimulation type. While both sham and active iTBS showed a negative relationship with corticomotor conductance potential, nMEPs associated with sham stimulation had lower nMEP amplitudes. Sham associated nMEPs also changed at a lower rate as the corticomotor conductance potential increased (p < 0.001, χ^2^ = 15.2). Consequently, as the corticomotor conductance potential approached zero, nMEP amplitudes were greater indicating a higher degree of excitation relative to sham (Additional file 1: Fig. S1A). There was an interaction between the corticomotor conductance potential and group (p < 0.001, χ^2^ = 13.3) suggesting that while this parameter has predictive potential across both groups, the exact correlation is group specific (Additional file 1: Fig. S1B). There was no difference in corticomotor conductance potential between groups (p = 0.89) (Table 2).

### Post hoc results

There was a relationship in the SCI group between RMT and nMEP (p < 0.001, χ^2^ = 7.7). There was no relationship between nMEPs and RMT in the nonimpaired group (p = 0.47, χ^2^ = 0.5).

## Discussion

The primary objective of this study was to determine the effect of iTBS on the corticomotor excitability of the biceps as measured by MEPs in response to TMS in individuals with tetraplegia and nonimpaired individuals. A secondary objective was to assess the reproducibility of iTBS effects across three sessions. We hypothesized that in both subject groups, biceps corticomotor excitability (i.e., normalized MEPs) would be increased following active iTBS relative to baseline, and biceps corticomotor excitability would be unchanged following sham iTBS relative to baseline. This hypothesis was supported in the SCI group; there was an increase in nMEP amplitude after active iTBS relative to sham. This hypothesis was not supported in the nonimpaired group; there was no change in biceps nMEPs after either active or sham iTBS. These findings suggest that iTBS has more homogeneous facilitatory effects in the biceps in individuals with incomplete tetraplegia than nonimpaired individuals, likely due to changes in corticomotor control after motor function loss.

The results from this study reinforce that corticomotor excitability is modifiable with iTBS in individuals with tetraplegia. This supports the modifiability findings from Fassett et al. in which iTBS was targeted to the flexor carpi radialis in individuals with tetraplegia [9]. While their results showed MEP reduction following iTBS, this could be due to differences in the targeted cortical motor region, or other factors influencing responses to iTBS. Previous studies have indicated that changes induced by iTBS in nonimpaired individuals depend on the cortical region targeted due to inherent differences in corticospinal control among muscles [32]. The findings from this study suggest that this may also be true for individuals with SCI, which could be further affected by the degree of damage to a muscle’s corticospinal tracts after injury, which is non-uniform after SCI [44].

Our results suggest that individuals with SCI exhibit a more homogeneous facilitatory response to iTBS targeting the biceps than nonimpaired individuals. In contrast to the nonimpaired group, the more uniform response of the SCI group may be the result of neuroplastic changes that occur post injury. For instance, the post-SCI system exhibits reduced intracortical inhibition and therefore greater neuroplastic response from disinhibition of gamma-Aminobutyric acid (GABA) transmitting interneurons to compensate for the loss of corticospinal axons [45]. Additionally, corticomotor plasticity can make alternate use of neural circuits that no longer have a functional muscular target available, as cortical map representation of nonparalyzed or less paralyzed muscle increases at the expense of paralyzed muscle [46]. This process can be facilitated by electrical stimulation along the corticomotor pathway; reactivation of neural circuits has been demonstrated after noninvasive electrical spinal neuromodulation in individuals with SCI, making them more responsive to facilitatory techniques, such as those for bladder control [47]. These results in spinal stimulation are relevant to our results in cortical stimulation because below-injury reorganization enhances excitability of motor pathways, reflective of cortical motor representation changes [48], and reorganization occurs above injury in the cortical projection system [49]. Corticospinal neurons projecting to the hand can branch to the arm which can improve voluntary upper limb movement of retained functional regions after reorganization [46]. Finally, while the lack of an effect of iTBS in the nonimpaired group was unexpected because meta-analysis suggests that iTBS is regarded as excitatory when targeting distal hand muscles, responsiveness has been seen to vary across individuals and within repeated sessions of the same individual [8, 13, 17, 50].

Within both the SCI and nonimpaired groups, there was a significant interaction between the corticomotor conductance potential and stimulation type (active or sham), which demonstrated that individuals presenting with lower ratios were more responsive to active iTBS than those with higher ratios. While there was a groupwide response to iTBS in our SCI group, the interaction of group with corticomotor conductance potential suggests that the magnitude of the response may be predictable. For individuals with tetraplegia, low ratios may indicate that the corticospinal tract of the muscle has potential to increase its conductance from iTBS, while high ratios could indicate that the corticospinal tract of the muscle is less likely to respond to iTBS. This interaction between the corticomotor conductance potential and type of stimulation was similarly found in the nonimpaired group. Thus, the corticomotor conductance potential could be used as a predictive measure of an individual’s responsiveness to iTBS. Future studies should investigate motor threshold changes as a potential effect of iTBS.

Our results further highlight the differences in corticomotor excitability between the nonimpaired and SCI populations, the effect of the corticomotor conductance potential, and how these population differences can affect the response to iTBS. While proximal muscles of the upper limb are likely to be less impaired relative to distal muscles after SCI, these muscles cannot necessarily be considered analogous to nonimpaired muscles [44]. This is demonstrated by our findings that the baseline nMEPs are higher in the SCI group relative to the nonimpaired group, which is consistent with other studies [48, 51]. Furthermore, while the groups respond within different regions of the corticomotor conductance potential profile, this work indicates that the corticomotor conductance potential has viability for predicting the effect of iTBS in both groups, despite the various neuroplastic changes that occur after SCI.

We assessed how corticomotor conductance potential affected nMEPs and the efficacy of iTBS in either nonimpaired individuals and those with SCI. We hypothesized that corticomotor excitability, as measured by nMEPs, would relate to the interaction between corticomotor conductance potential and stimulation type (i.e. active or sham iTBS). This hypothesis was supported; the motor threshold ratio was found to be negatively correlated with nMEPs and had a significant interaction between coil type.

### Limitations

This study used a single stimulator to represent the clinical environment in which iTBS may be delivered and MEPs assessed with the same device. However, most of our participants had RMT values ≥ 84% MSO. Thus, we could not assess biceps corticomotor excitability (i.e., MEPs) at stimulus intensities of 120% RMT resulting in potential under-stimulation and greater MEP variability [36]. This potential limitation could be addressed by using a monophasic stimulator to evaluate RMTs and collect MEPs. RMT of the biceps when determined by a monophasic stimulator are typically 50–60%MSO [34]. We evaluated the relationship between nMEPs and RMT to determine if under-stimulation influenced our results and found no correlation in the nonimpaired group, but there was a correlation in the SCI group. However, despite recording MEPs at less than 120% of RMT in many of the SCI subjects, the effect of iTBS was still significantly faciliatory in the SCI group as a whole (i.e., MEPs increased after iTBS relative to baseline). Another potential limitation is that sham stimulation was always delivered prior to active stimulation. While this was done to prevent any response to active stimulation biasing the response to sham within the same session, we cannot exclude the possibility of an order effect. It is also possible that effects of iTBS within the first 10 min were not captured due to the 10 min interval schedule of MEP elicitation that was chosen based on previous work targeting other muscles [8, 13, 52]. The time frame was chosen for relevance as an adjunct to rehabilitation protocols which would begin a few minutes after iTBS priming. Also, in some iTBS sessions of our SCI group, AMT was greater than 72% MSO, although this was an exclusion criterion of the first session. AMT greater than 72% MSO would dictate an iTBS intensity of greater than 57% MSO, whereas safety limitations in our stimulator imposed by the manufacturer held maximum iTBS intensity to 57% MSO. In these individuals, 57% MSO was used for their iTBS, and potential under-stimulation during iTBS delivery was still insufficient to obscure the effect of iTBS in this SCI group. Finally, as the sample size is limited, our results should be confirmed in a larger clinical trial.

## Conclusions

The biceps brachii is a responsive target for iTBS to increase corticomotor excitability in individuals with tetraplegia, emphasizing the potential of iTBS as an adjunct to physical therapy for motor rehabilitation. Furthermore, our comparison with the nonimpaired group provides evidence for differences in effects of iTBS between nonimpaired and SCI groups suggesting that neuroplastic changes after SCI play a role in the neuromodulation susceptibility of a motor cortical target. Therefore, further research is needed to confirm our preliminary findings in a larger clinical trial, investigate how muscle target and injury level influence effects of iTBS, and establish the amount of corticomotor excitability change that is needed to affect rehabilitation outcomes and functional ability.

## Supplementary Information

**Additional file 1: Table S1.** Motor thresholds by session in SCI participants prior to iTBS presented as percent maximum stimulator output (%MSO). **Table S2.** Motor thresholds by session in the nonimpaired participants prior to iTBS presented as percent maximum stimulator output (%MSO). **Table S3.** Maximum Voluntary Contraction (MVC) EMG value by session in both groups prior to iTBS. **Figure S1.** Interaction Between Corticomotor Conductance Potential (AMT/RMT) and Group, and their Effect on Modeled nMEPs.

## Data Availability

Data is available through the Open Science Framework – https://osf.io/za78p/?view_only=1f23b70066d64faba087b2b4c0784baa

## Abbreviations

AMT: Active motor threshold
EMG: Electromyography
GABA: Gamma-Aminobutyric acid
ISNCSCI: International Standards for Neurological Classification of Spinal Cord Injury
iTBS: Intermittent theta burst stimulation
LMEM: Linear mixed effects model
M1: Primary motor cortex
MEP: Motor evoked potential
MSO: Maximum stimulator output
MVC: Maximum voluntary contraction
nMEP: Normalized motor evoked potential
RMS: Root Mean Squared
RMT: Resting motor threshold
rTMS: Repetitive transcranial magnetic stimulation
SCI: Spinal cord injury
TMS: Transcranial magnetic stimulation
TBS: Theta burst stimulation
