# Supplementary material for: Intermittent theta burst stimulation modulates biceps brachii corticomotor excitability in individuals with tetraplegia
Source: J Neuroeng Rehabil. 2022 Jul 17;19:73. doi: 10.1186/s12984-022-01049-9 (PMC9290267; doi:10.1186/s12984-022-01049-9)
Supplement: Supplementary file 1 — Additional file 1: Table S1. Motor thresholds by session in SCI participants prior to iTBS presented as percent maximum stimulator output (%MSO). Table S2. Motor thresholds by session in the nonimpaired participants prior to iTBS presented as percent maximum stimulator output (%MSO). Table S3. Maximum Voluntary Contraction (MVC) EMG value by session in both groups prior to iTBS. Figure S1. Interaction Between Corticomotor Conductance Potential (AMT/RMT) and Group, and their Effect on Modeled nMEPs. [file 12984_2022_1049_MOESM1_ESM.docx]

**Table S1. Motor thresholds by session in SCI participants prior to iTBS presented as percent maximum stimulator output (%MSO).**

|  | Session 1 | | | | Session 2 | | | | Session 3 | | | |
| --- | --- | --- | --- | --- | --- | --- | --- | --- | --- | --- | --- | --- |
| Participant | Biceps RMT^a^ Prior to Sham iTBS | Biceps AMT^b^ Prior to Sham iTBS | Biceps RMT Prior to Active iTBS | Biceps AMT Prior to Active iTBS | Biceps RMT^a^ Prior to Sham iTBS | Biceps AMT^b^ Prior to Sham iTBS | Biceps RMT Prior to Active iTBS | Biceps AMT Prior to Active iTBS | Biceps RMT^a^ Prior to Sham iTBS | Biceps AMT^b^ Prior to Sham iTBS | Biceps RMT Prior to Active iTBS | Biceps AMT Prior to Active iTBS |
| 01 | 95 | 57 | 95 | 51 | 84 | 47 | 92 | 34 | 98 | 47 | 97 | 47 |
| 02 | 80 | 34 | 82 | 53 | 100 | 20 | 80 | 41 | 88 | 45 | 88 | 59 |
| 03 | 81 | 53 | 77 | 58 | 85 | 55 | 80 | 54 | 98 | 53 | 91 | 42 |
| 04 | 93 | 74 | 95 | 74 | 100 | 100 | 94 | 79 | 90 | 75 | 97 | 79 |
| 05 | 100 | 68 | 100 | 69 | 100 | 70 | 100 | 80 | 100 | 56 | 100 | 66 |
| 06 | 100 | 100 | 100 | 100 | 100 | 100 | 100 | 100 | 100 | 100 | 100 | 94 |
| 07 | 100 | 67 | 100 | 70 | 100 | 57 | 100 | 66 | 100 | 51 | 100 | 48 |
| 08 | 67 | 43 | 71 | 45 | 57 | 53 | 66 | 56 | 65 | 50 | 81 | 53 |
| 09 | 100 | 70 | 87 | 59 | 87 | 61 | 91 | 63 | 91 | 66 | 100 | 79 |
| 10 | 100 | 100 | 100 | 100 | 100 | 93 | 100 | 88 | 100 | 100 | 100 | 100 |

a) RMT: resting motor threshold as percent of maximum stimulator output (% MSO) measured with biphasic PA/AP stimulation; b) AMT: active motor threshold as % MSO measured with biphasic PA/AP stimulation.

**Table S2. Motor thresholds by session in the nonimpaired participants prior to iTBS presented as percent maximum stimulator output (%MSO).**

|  | Session 1 | | | | Session 2 | | | | Session 3 | | | |
| --- | --- | --- | --- | --- | --- | --- | --- | --- | --- | --- | --- | --- |
| Participant | Biceps RMT^a^ Prior to Sham iTBS | Biceps AMT^b^ Prior to Sham iTBS | Biceps RMT Prior to Active iTBS | Biceps AMT Prior to Active iTBS | Biceps RMT^a^ Prior to Sham iTBS | Biceps AMT^b^ Prior to Sham iTBS | Biceps RMT Prior to Active iTBS | Biceps AMT Prior to Active iTBS | Biceps RMT^a^ Prior to Sham iTBS | Biceps AMT^b^ Prior to Sham iTBS | Biceps RMT Prior to Active iTBS | Biceps AMT Prior to Active iTBS |
| 01 | 90 | 52 | 100 | 61 | 100 | 48 | 100 | 56 | 100 | 60 | 100 | 51 |
| 02 | 77 | 56 | 83 | 56 | 85 | 50 | 88 | 60 | 95 | 65 | 88 | 54 |
| 03 | 100 | 68 | 87 | 60 | 61 | 53 | 67 | 56 | 61 | 58 | 59 | 60 |
| 04 | 100 | 71 | 100 | 69 | 90 | 67 | 100 | 74 | 96 | 68 | 98 | 59 |
| 05 | 84 | 56 | 85 | 64 | 86 | 63 | 95 | 54 | 85 | 56 | 74 | 44 |
| 06 | 85 | 53 | 83 | 49 | 97 | 60 | 95 | 67 | 77 | 42 | 88 | 42 |
| 07 | 95 | 68 | 100 | 59 | 100 | 46 | 100 | 55 | 100 | 45 | 100 | 34 |
| 08 | 88 | 62 | 74 | 52 | 83 | 66 | 85 | 61 | 100 | 54 | 83 | 58 |
| 09 | 100 | 67 | 100 | 68 | 100 | 72 | 100 | 57 | 100 | 60 | 100 | 53 |
| 10 | 76 | 50 | 78 | 60 | 74 | 60 | 76 | 60 | 70 | 43 | 71 | 45 |

a) RMT: resting motor threshold as percent of maximum stimulator output (% MSO) measured with biphasic PA/AP stimulation; b) AMT: active motor threshold as % MSO measured with biphasic PA/AP stimulation.

**Table S3. Maximum Voluntary Contraction (MVC) EMG value by session in both groups prior to iTBS.**

| Participant | Group | Session 1 MVC^a^ | Session 2 MVC | Session 3 MVC |
| --- | --- | --- | --- | --- |
| 01 | NI^b^ | 228.3 | 100.0 | 239.5 |
| 02 | NI | 204.8 | 85.0 | 240.3 |
| 03 | NI | 96.4 | 61.0 | 96.0 |
| 04 | NI | 162.0 | 90.0 | 210.6 |
| 05 | NI | 442.6 | 86.0 | 354.9 |
| 06 | NI | 392.5 | 97.0 | 527.7 |
| 07 | NI | 281.8 | 100.0 | 446.2 |
| 08 | NI | 233.7 | 83.0 | 183.3 |
| 09 | NI | 339.6 | 100.0 | 329.5 |
| 10 | NI | 143.7 | 74.0 | 206.9 |
| 01 | SCI^c^ | 418.3 | 607.7 | 638.2 |
| 02 | SCI | 454.0 | 495.4 | 431.6 |
| 03 | SCI | 123.9 | 235.2 | 385.3 |
| 04 | SCI | 99.1 | 80.1 | 96.8 |
| 05 | SCI | 368.4 | 276.1 | 270.6 |
| 06 | SCI | 55.1 | 42.5 | 61.1 |
| 07 | SCI | 275.0 | 285.1 | 282.2 |
| 08 | SCI | 277.2 | 129.8 | 322.3 |
| 09 | SCI | 70.7 | 73.5 | 108.8 |
| 10 | SCI | 45.2 | 57.1 | 55.3 |

a) MVC: maximum voluntary contraction (mV); b) NI: nonimpaired group; c) SCI: spinal cord injury group.


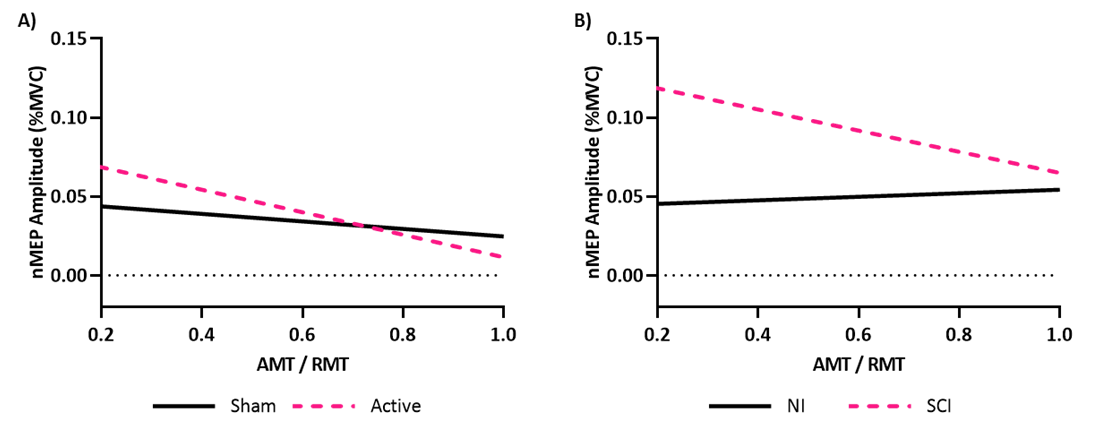


**Figure S1. Interaction Between Corticomotor Conductance Potential (AMT/RMT) and Group, and their Effect on Modeled nMEPs.**

A) There was a negative correlation between corticomotor conductance potential and nMEP amplitude that differed by stimulation type suggesting that at lower corticomotor conductance potentials, individuals in either group are more responsive to iTBS. (B) The effect of corticomotor conductance potential on nMEP amplitude was unique by group across both stimulation types suggesting that low corticomotor conductance potentials were associated with greater disparity in stimulation efficacy between the groups.
